# Crystal structure of dimethyl 2,5-bis­[(di­phen­oxy­phosphor­yl)­oxy]cyclo­hexa-1,4-diene-1,4-di­carboxyl­ate

**DOI:** 10.1107/S2056989015008658

**Published:** 2015-05-13

**Authors:** Lei Gao, Zongshan Ma, Hong Yan

**Affiliations:** a College of Life Science and Bio-engineering, Beijing University of Technology, Pingleyuan Street No. 100, Chaoyang District, Beijing 100124, People’s Republic of China

**Keywords:** crystal structure, cyclo­hexa-1,4-diene, C—H⋯O hydrogen bonds

## Abstract

In the title compound, C_34_H_30_O_12_P_2_, which was synthesized *via* the esterification of dimethyl 2,5-dioxo-1,4-cyclo­hexa­nedi­carboxyl­ate with diphenyl chloro­phosphate, the mol­ecule has crystallographic inversion symmetry. The dihedral angles between the plane of the cyclo­hexa-1,4-diene ring and those of the two benzene rings of the substituent phosphate groups are 41.0 (1) and 89.5 (1)°, while that with the ester group is 3.1 (3)°. In the crystal, only weak inter­molecular C—H⋯O hydrogen bonds are present.

## Related literature   

For background information on cyclo­hexa-1,4-dienes, see: El-Rayyes & Al-Hajjar (1978 ▸). For the synthesis of the title compound, see: Chaignaud *et al.* (2008 ▸).
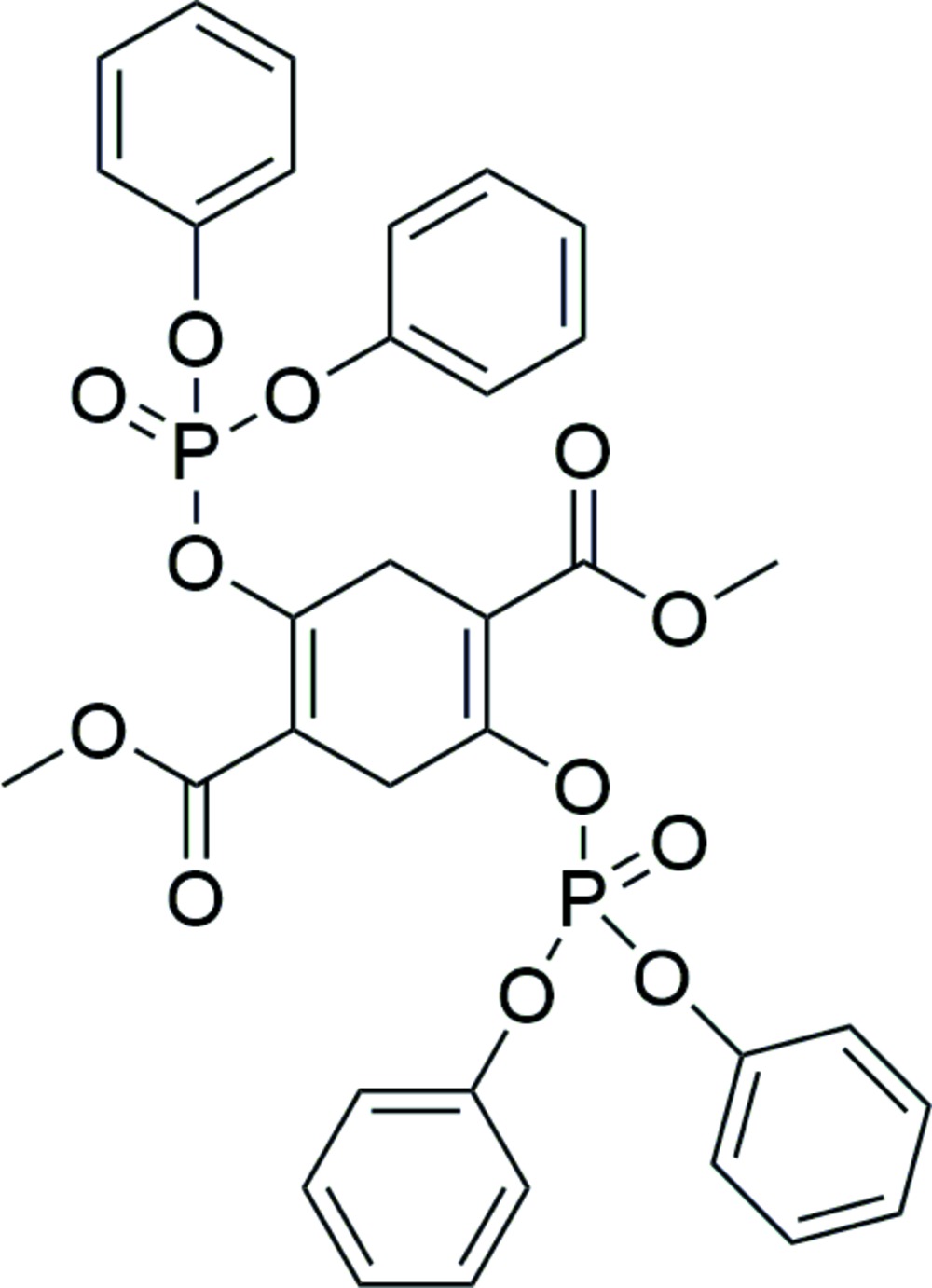


## Experimental   

### Crystal data  

C_34_H_30_O_12_P_2_
*M* *_r_* = 692.52
Monoclinic, 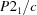

*a* = 12.272 (10) Å
*b* = 10.629 (8) Å
*c* = 13.174 (10) Å
β = 113.644 (10)°
*V* = 1574 (2) Å^3^
*Z* = 2
Mo *K*α radiation
μ = 0.21 mm^−1^
*T* = 113 K
0.20 × 0.18 × 0.12 mm

### Data collection  

Rigaku Saturn724 CCD diffractometer
Absorption correction: multi-scan (*CrystalClear*; Rigaku, 2005 ▸) *T*_min_ = 0.960, *T*_max_ = 0.976
15948 measured reflections
3758 independent reflections
2264 reflections with *I* > 2σ(*I*)
*R*_int_ = 0.100

### Refinement  

*R*[*F* ^2^ > 2σ(*F* ^2^)] = 0.045
*wR*(*F* ^2^) = 0.099
*S* = 1.00
3758 reflections
218 parameters
H-atom parameters constrained
Δρ_max_ = 0.54 e Å^−3^
Δρ_min_ = −0.62 e Å^−3^

### 

Data collection: *CrystalClear* (Rigaku, 2005 ▸); cell refinement: *CrystalClear*; data reduction: *CrystalClear*; program(s) used to solve structure: *SHELXS97* (Sheldrick, 2008 ▸); program(s) used to refine structure: *SHELXL97* (Sheldrick, 2008 ▸); molecular graphics: *SHELXTL* (Sheldrick, 2008 ▸); software used to prepare material for publication: *CrystalStructure* (Rigaku, 2005 ▸).

## Supplementary Material

Crystal structure: contains datablock(s) I, New_Global_Publ_Block. DOI: 10.1107/S2056989015008658/zs2331sup1.cif

Structure factors: contains datablock(s) I. DOI: 10.1107/S2056989015008658/zs2331Isup2.hkl

**Fig. 1.**
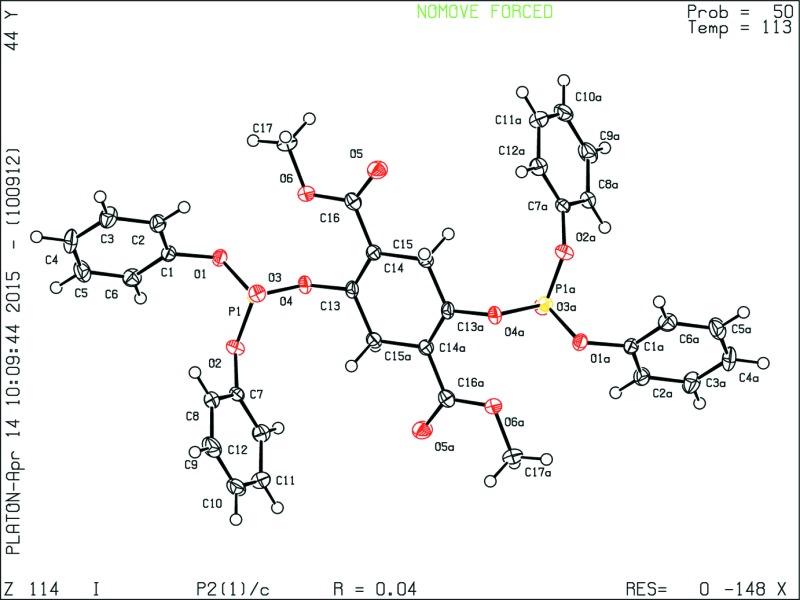
The molecular conformation and atom numbering scheme for the title compound, with probability ellipsoids drawn at the 50% level. For symmetry code (a): -x, -y + 1, -z.

Supporting information file. DOI: 10.1107/S2056989015008658/zs2331Isup3.cml

x y z . DOI: 10.1107/S2056989015008658/zs2331fig1.tif
The mol­ecular conformation and atom numbering scheme for the title compound, with probability ellipsoids drawn at the 50% level. For symmetry code (a): −*x*, −*y* + 1, −*z*.

**Fig. 2.**
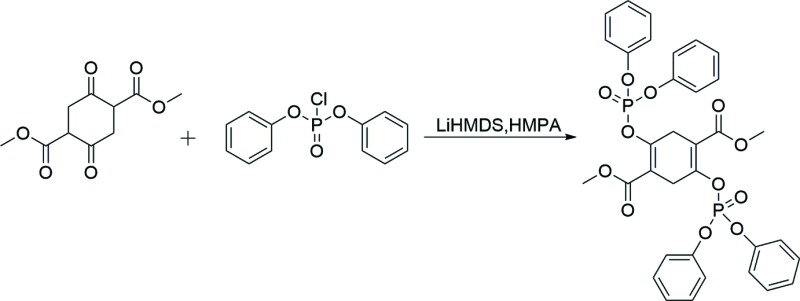
Synthetic route for the title compound.

. DOI: 10.1107/S2056989015008658/zs2331fig2.tif
Synthetic route for the title compound.

CCDC reference: 1057648

Additional supporting information:  crystallographic information; 3D view; checkCIF report

## Figures and Tables

**Table 1:** Hydrogen-bond geometry (, )

*D*H*A*	*D*H	H*A*	*D* *A*	*D*H*A*
C6H6O3^i^	0.95	2.50	3.345(4)	148
C9H9O5^ii^	0.95	2.59	3.405(4)	144
C10H10O3^iii^	0.95	2.46	3.381(4)	163
C15H15*B*O1^iv^	0.99	2.56	3.409(4)	144

Symmetry codes: (i) 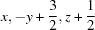; (ii) 
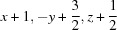
; (iii) 
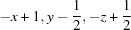
; (iv) 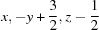.

## supplementary crystallographic information

### S1. Comment

1,4-Cyclohexadiene is a useful and fundamental structural motif found in a wide range of organic materials and biologically active molecules (El-Rayyes & Al-Hajjar, 1978). The synthetic routes for the preparation of derivatives of this parent compound have been reported (Chaignaud *et al.*, 2008) but their crystal structures were not described.

The title compound, C_34_H_30_O_12_P_2_, was synthesized by the esterification of dimethyl 2,5-dioxo-1,4-cyclohexanedicarboxylate with diphenyl chlorophosphate using the reported procedure of Chaignaud *et al.* (2008) and the structure is reported herein.

The molecule of the title compound has crystallographic inversion symmetry (Fig. 1), with dihedral angles between the cyclohexa-1,4-diene ring and the two benzene rings of the substituent phosphate group of 41.0 (1) [C1–C6] and 89.5 (1)° [C7–C12]. The ester group is essentially coplanar with the cyclohexadiene group [dihedral angle = 3.1 (3)°]. In the crystal, only weak intermolecular C—H···O hydrogen bonds are present (Table 1).

### S2. Experimental

The title compound was synthesized using the basic procedure of Chaignaud *et al.* (2008) (Fig. 2), as follows: A solution of LiHMDS (1 M in THF, 7.9 mL) in THF (20 mL) was cooled to -78 °C under nitrogen. Subsequently, a mixture of dimethyl 2,5-dioxo-1,4-cyclohexanedicarboxylate (3.19 mmol), diphenyl chlorophosphate (6.67 mmol) and HMPA (8.90 mmol) in anhydrous THF (5 mL) were added dropwise over 5 min. The mixture was stirred at -78 °C for 1h under nitrogen and after completion of the reaction, the mixture was diluted with water (30 mL) and extracted with ethyl acetate (3×15 mL), dried with anhydrous MgSO_4_ and filtered. Subsequently, the product obtained by evaporation of the solvent was recrystallized from ethyl acetate giving a white solid in 10% yield (m.p. 118–120 °C).

### S3. Refinement

H atoms were were positioned geometrically and refined using a riding model with C—H = 0.95–0.99 Å and *U*_iso_(H) = 1.2*U*_eq_(C) (aromatic) or 1.5*U*_eq_(C) (methyl).

### Crystal data

**Table:**

C_34_H_30_O_12_P_2_	*F*(000) = 720
*M**_r_* = 692.52	*D*_x_ = 1.461 Mg m^−^^3^
Monoclinic, *P*2_1_/*c*	Melting point = 391–393 K
Hall symbol: -P 2ybc	Mo *K*α radiation, λ = 0.71073 Å
*a* = 12.272 (10) Å	Cell parameters from 5446 reflections
*b* = 10.629 (8) Å	θ = 1.7–28.3°
*c* = 13.174 (10) Å	µ = 0.21 mm^−^^1^
β = 113.644 (10)°	*T* = 113 K
*V* = 1574 (2) Å^3^	Prism, colorless
*Z* = 2	0.20 × 0.18 × 0.12 mm

### Data collection

**Table:**

Rigaku Saturn724 CCD diffractometer	3758 independent reflections
Radiation source: rotating anode	2264 reflections with *I* > 2σ(*I*)
Multilayer monochromator	*R*_int_ = 0.100
Detector resolution: 14.22 pixels mm^-1^	θ_max_ = 28.0°, θ_min_ = 1.8°
ω and φ scans	*h* = −16→16
Absorption correction: multi-scan (*CrystalClear*; Rigaku, 2005)	*k* = −13→13
*T*_min_ = 0.960, *T*_max_ = 0.976	*l* = −17→17
15948 measured reflections	

### Refinement

**Table:**

Refinement on *F*^2^	Primary atom site location: structure-invariant direct methods
Least-squares matrix: full	Secondary atom site location: difference Fourier map
*R*[*F*^2^ > 2σ(*F*^2^)] = 0.045	Hydrogen site location: inferred from neighbouring sites
*wR*(*F*^2^) = 0.099	H-atom parameters constrained
*S* = 1.00	*w* = 1/[σ^2^(*F*_o_^2^) + (0.012*P*)^2^] where *P* = (*F*_o_^2^ + 2*F*_c_^2^)/3
3758 reflections	(Δ/σ)_max_ = 0.002
218 parameters	Δρ_max_ = 0.54 e Å^−^^3^
0 restraints	Δρ_min_ = −0.62 e Å^−^^3^

### Special details

**Table:**

Geometry. All e.s.d.'s (except the e.s.d. in the dihedral angle between two l.s. planes) are estimated using the full covariance matrix. The cell e.s.d.'s are taken into account individually in the estimation of e.s.d.'s in distances, angles and torsion angles; correlations between e.s.d.'s in cell parameters are only used when they are defined by crystal symmetry. An approximate (isotropic) treatment of cell e.s.d.'s is used for estimating e.s.d.'s involving l.s. planes.
Refinement. Refinement of *F*^2^ against ALL reflections. The weighted *R*-factor *wR* and goodness of fit *S* are based on *F*^2^, conventional *R*-factors *R* are based on *F*, with *F* set to zero for negative *F*^2^. The threshold expression of *F*^2^ > σ(*F*^2^) is used only for calculating *R*-factors(gt) *etc*. and is not relevant to the choice of reflections for refinement. *R*-factors based on *F*^2^ are statistically about twice as large as those based on *F*, and *R*- factors based on ALL data will be even larger.

### Fractional atomic coordinates and isotropic or equivalent isotropic displacement parameters (Å^2^)

**Table:**

	*x*	*y*	*z*	*U*_iso_*/*U*_eq_	
P1	0.17401 (5)	0.72276 (5)	0.24500 (4)	0.01714 (15)	
O1	0.12748 (11)	0.81381 (12)	0.31240 (10)	0.0189 (3)	
O2	0.26861 (12)	0.64338 (12)	0.34149 (11)	0.0211 (3)	
O3	0.21532 (12)	0.77428 (12)	0.16508 (10)	0.0194 (3)	
O4	0.06126 (12)	0.63451 (12)	0.19634 (10)	0.0187 (3)	
O5	−0.16106 (13)	0.79217 (13)	−0.10725 (11)	0.0285 (4)	
O6	−0.06056 (12)	0.82652 (13)	0.07360 (11)	0.0259 (4)	
C1	0.20189 (18)	0.91573 (18)	0.36916 (16)	0.0181 (5)	
C2	0.20083 (19)	1.02272 (19)	0.30979 (17)	0.0229 (5)	
H2	0.1545	1.0266	0.2323	0.027*	
C3	0.2684 (2)	1.1239 (2)	0.36526 (18)	0.0288 (6)	
H3	0.2690	1.1986	0.3259	0.035*	
C4	0.3353 (2)	1.1172 (2)	0.47791 (18)	0.0310 (6)	
H4	0.3818	1.1873	0.5159	0.037*	
C5	0.3350 (2)	1.0088 (2)	0.53572 (18)	0.0298 (6)	
H5	0.3816	1.0046	0.6132	0.036*	
C6	0.26714 (19)	0.90613 (19)	0.48111 (17)	0.0237 (5)	
H6	0.2659	0.8313	0.5201	0.028*	
C7	0.37059 (18)	0.58701 (19)	0.33647 (16)	0.0193 (5)	
C8	0.45474 (17)	0.6591 (2)	0.31964 (15)	0.0221 (5)	
H8	0.4432	0.7469	0.3063	0.027*	
C9	0.55660 (19)	0.6006 (2)	0.32263 (17)	0.0294 (6)	
H9	0.6158	0.6483	0.3105	0.035*	
C10	0.5728 (2)	0.4722 (2)	0.34328 (17)	0.0317 (6)	
H10	0.6431	0.4326	0.3455	0.038*	
C11	0.4874 (2)	0.4024 (2)	0.36046 (17)	0.0290 (6)	
H11	0.4987	0.3147	0.3747	0.035*	
C12	0.38516 (19)	0.46028 (19)	0.35698 (17)	0.0247 (5)	
H12	0.3256	0.4128	0.3687	0.030*	
C13	0.02855 (17)	0.57494 (18)	0.09354 (16)	0.0173 (5)	
C14	−0.04157 (18)	0.62776 (18)	−0.00080 (16)	0.0167 (5)	
C15	−0.07850 (18)	0.55461 (17)	−0.10704 (15)	0.0186 (5)	
H15A	−0.1664	0.5490	−0.1412	0.022*	
H15B	−0.0532	0.6015	−0.1588	0.022*	
C16	−0.09356 (18)	0.75651 (19)	−0.01748 (16)	0.0196 (5)	
C17	−0.11425 (19)	0.95035 (18)	0.05728 (18)	0.0302 (6)	
H17A	−0.0905	0.9985	0.0061	0.045*	
H17B	−0.0876	0.9943	0.1286	0.045*	
H17C	−0.2011	0.9420	0.0262	0.045*	

### Atomic displacement parameters (Å^2^)

**Table:**

	*U*^11^	*U*^22^	*U*^33^	*U*^12^	*U*^13^	*U*^23^
P1	0.0148 (3)	0.0166 (3)	0.0186 (3)	−0.0009 (2)	0.0052 (2)	−0.0017 (2)
O1	0.0177 (8)	0.0178 (8)	0.0225 (8)	−0.0025 (6)	0.0093 (7)	−0.0050 (6)
O2	0.0168 (8)	0.0239 (8)	0.0207 (8)	0.0035 (7)	0.0056 (7)	0.0029 (6)
O3	0.0193 (8)	0.0208 (8)	0.0194 (7)	−0.0011 (6)	0.0090 (7)	0.0005 (6)
O4	0.0166 (7)	0.0197 (8)	0.0182 (8)	−0.0054 (6)	0.0055 (6)	−0.0040 (6)
O5	0.0344 (9)	0.0248 (9)	0.0226 (8)	0.0067 (7)	0.0078 (8)	0.0036 (7)
O6	0.0235 (8)	0.0227 (8)	0.0258 (9)	0.0062 (7)	0.0038 (7)	−0.0043 (7)
C1	0.0165 (11)	0.0177 (11)	0.0200 (11)	−0.0006 (9)	0.0070 (9)	−0.0040 (9)
C2	0.0272 (13)	0.0221 (12)	0.0168 (11)	0.0012 (10)	0.0060 (10)	−0.0016 (9)
C3	0.0354 (14)	0.0208 (13)	0.0301 (13)	−0.0050 (11)	0.0131 (12)	−0.0028 (10)
C4	0.0334 (14)	0.0261 (14)	0.0310 (14)	−0.0123 (11)	0.0103 (12)	−0.0132 (11)
C5	0.0276 (13)	0.0379 (15)	0.0179 (12)	−0.0024 (11)	0.0029 (10)	−0.0045 (10)
C6	0.0271 (13)	0.0225 (13)	0.0217 (12)	0.0013 (10)	0.0101 (10)	0.0023 (10)
C7	0.0143 (11)	0.0238 (12)	0.0158 (11)	0.0044 (9)	0.0017 (9)	−0.0044 (9)
C8	0.0171 (11)	0.0216 (12)	0.0237 (12)	−0.0007 (10)	0.0041 (10)	−0.0038 (9)
C9	0.0161 (12)	0.0398 (15)	0.0307 (14)	−0.0035 (11)	0.0077 (11)	−0.0036 (11)
C10	0.0184 (12)	0.0442 (16)	0.0264 (13)	0.0096 (12)	0.0026 (11)	−0.0099 (11)
C11	0.0289 (13)	0.0248 (13)	0.0296 (13)	0.0069 (11)	0.0077 (11)	−0.0034 (11)
C12	0.0216 (12)	0.0228 (13)	0.0275 (12)	−0.0006 (10)	0.0074 (10)	−0.0010 (10)
C13	0.0147 (10)	0.0186 (11)	0.0189 (11)	−0.0052 (9)	0.0071 (9)	−0.0058 (9)
C14	0.0144 (10)	0.0169 (11)	0.0188 (11)	−0.0012 (9)	0.0066 (9)	−0.0009 (9)
C15	0.0154 (11)	0.0201 (12)	0.0182 (11)	−0.0005 (9)	0.0046 (9)	−0.0002 (9)
C16	0.0158 (11)	0.0240 (13)	0.0199 (11)	−0.0025 (9)	0.0079 (10)	0.0003 (9)
C17	0.0270 (13)	0.0246 (13)	0.0355 (14)	0.0094 (11)	0.0088 (12)	−0.0039 (11)

### Geometric parameters (Å, º)

**Table:**

P1—O1	1.5677 (19)	C11—C12	1.382 (4)
P1—O2	1.5785 (19)	C13—C14	1.320 (3)
P1—O3	1.4469 (19)	C13—C15^i^	1.489 (3)
P1—O4	1.579 (2)	C14—C15	1.503 (3)
O1—C1	1.421 (3)	C14—C16	1.489 (3)
O2—C7	1.413 (3)	C2—H2	0.9500
O4—C13	1.400 (3)	C3—H3	0.9500
O5—C16	1.201 (3)	C4—H4	0.9500
O6—C16	1.330 (3)	C5—H5	0.9500
O6—C17	1.449 (3)	C6—H6	0.9500
C1—C2	1.377 (3)	C8—H8	0.9500
C1—C6	1.371 (3)	C9—H9	0.9500
C2—C3	1.376 (3)	C10—H10	0.9500
C3—C4	1.380 (3)	C11—H11	0.9500
C4—C5	1.382 (3)	C12—H12	0.9500
C5—C6	1.386 (3)	C15—H15A	0.9900
C7—C8	1.374 (3)	C15—H15B	0.9900
C7—C12	1.371 (3)	C17—H17A	0.9800
C8—C9	1.383 (3)	C17—H17B	0.9800
C9—C10	1.390 (3)	C17—H17C	0.9800
C10—C11	1.375 (4)		
			
O1—P1—O2	101.02 (7)	O5—C16—C14	121.40 (18)
O1—P1—O3	119.47 (8)	O6—C16—C14	115.08 (17)
O1—P1—O4	97.92 (8)	C1—C2—H2	121.00
O2—P1—O3	115.41 (9)	C3—C2—H2	121.00
O2—P1—O4	104.54 (8)	C2—C3—H3	120.00
O3—P1—O4	115.79 (8)	C4—C3—H3	120.00
P1—O1—C1	117.75 (13)	C3—C4—H4	120.00
P1—O2—C7	124.62 (13)	C5—C4—H4	120.00
P1—O4—C13	121.73 (14)	C4—C5—H5	120.00
C16—O6—C17	114.76 (16)	C6—C5—H5	120.00
O1—C1—C2	118.23 (17)	C1—C6—H6	121.00
O1—C1—C6	118.96 (17)	C5—C6—H6	121.00
C2—C1—C6	122.74 (19)	C7—C8—H8	121.00
C1—C2—C3	118.52 (19)	C9—C8—H8	121.00
C2—C3—C4	120.2 (2)	C8—C9—H9	120.00
C3—C4—C5	120.3 (2)	C10—C9—H9	120.00
C4—C5—C6	120.3 (2)	C9—C10—H10	120.00
C1—C6—C5	118.03 (19)	C11—C10—H10	120.00
O2—C7—C8	120.56 (18)	C10—C11—H11	120.00
O2—C7—C12	117.1 (2)	C12—C11—H11	120.00
C8—C7—C12	122.2 (2)	C7—C12—H12	120.00
C7—C8—C9	118.3 (2)	C11—C12—H12	120.00
C8—C9—C10	120.3 (2)	C14—C15—H15A	109.00
C9—C10—C11	120.2 (2)	C14—C15—H15B	109.00
C10—C11—C12	119.8 (2)	H15A—C15—H15B	108.00
C7—C12—C11	119.3 (2)	C13^i^—C15—H15A	109.00
O4—C13—C14	122.97 (18)	C13^i^—C15—H15B	109.00
O4—C13—C15^i^	110.99 (16)	O6—C17—H17A	109.00
C14—C13—C15^i^	125.99 (18)	O6—C17—H17B	109.00
C13—C14—C15	119.73 (18)	O6—C17—H17C	109.00
C13—C14—C16	127.52 (18)	H17A—C17—H17B	109.00
C15—C14—C16	112.75 (16)	H17A—C17—H17C	109.00
C13^i^—C15—C14	114.28 (16)	H17B—C17—H17C	109.00
O5—C16—O6	123.51 (19)		
			
O2—P1—O1—C1	75.66 (14)	C3—C4—C5—C6	−0.3 (4)
O3—P1—O1—C1	−52.09 (15)	C4—C5—C6—C1	0.4 (4)
O4—P1—O1—C1	−177.76 (12)	O2—C7—C8—C9	175.83 (17)
O1—P1—O2—C7	−151.58 (15)	C12—C7—C8—C9	0.6 (3)
O3—P1—O2—C7	−21.23 (17)	O2—C7—C12—C11	−175.66 (18)
O4—P1—O2—C7	107.15 (15)	C8—C7—C12—C11	−0.3 (3)
O1—P1—O4—C13	149.57 (14)	C7—C8—C9—C10	−0.6 (3)
O2—P1—O4—C13	−106.81 (14)	C8—C9—C10—C11	0.3 (3)
O3—P1—O4—C13	21.34 (17)	C9—C10—C11—C12	0.1 (3)
P1—O1—C1—C2	81.9 (2)	C10—C11—C12—C7	−0.1 (3)
P1—O1—C1—C6	−101.1 (2)	O4—C13—C14—C15	−176.33 (19)
P1—O2—C7—C8	59.5 (2)	O4—C13—C14—C16	2.7 (4)
P1—O2—C7—C12	−125.04 (17)	C15^i^—C13—C14—C15	0.7 (4)
P1—O4—C13—C14	−88.2 (2)	C15^i^—C13—C14—C16	179.7 (2)
P1—O4—C13—C15^i^	94.39 (18)	O4—C13—C15^i^—C14^i^	176.67 (18)
C17—O6—C16—O5	1.1 (3)	C14—C13—C15^i^—C14^i^	−0.7 (3)
C17—O6—C16—C14	−178.27 (19)	C13—C14—C15—C13^i^	−0.6 (3)
O1—C1—C2—C3	177.0 (2)	C16—C14—C15—C13^i^	−179.77 (19)
C6—C1—C2—C3	0.2 (4)	C13—C14—C16—O5	−176.4 (2)
O1—C1—C6—C5	−177.2 (2)	C13—C14—C16—O6	2.9 (4)
C2—C1—C6—C5	−0.4 (4)	C15—C14—C16—O5	2.7 (3)
C1—C2—C3—C4	0.0 (4)	C15—C14—C16—O6	−177.98 (19)
C2—C3—C4—C5	0.1 (4)		

Symmetry code: (i) −*x*, −*y*+1, −*z*.

### Hydrogen-bond geometry (Å, º)

**Table:**

*D*—H···*A*	*D*—H	H···*A*	*D*···*A*	*D*—H···*A*
C2—H2···O5^ii^	0.95	2.56	3.191 (4)	124
C6—H6···O3^iii^	0.95	2.50	3.345 (4)	148
C9—H9···O5^iv^	0.95	2.59	3.405 (4)	144
C10—H10···O3^v^	0.95	2.46	3.381 (4)	163
C15—H15*B*···O1^vi^	0.99	2.56	3.409 (4)	144

Symmetry codes: (ii) −*x*, −*y*+2, −*z*; (iii) *x*, −*y*+3/2, *z*+1/2; (iv) *x*+1, −*y*+3/2, *z*+1/2; (v) −*x*+1, *y*−1/2, −*z*+1/2; (vi) *x*, −*y*+3/2, *z*−1/2.
