# Supplementary figures and images for: Crystal structure of dimethyl 2,5-bis­[(di­phen­oxy­phosphor­yl)­oxy]cyclo­hexa-1,4-diene-1,4-di­carboxyl­ate
Source: Acta Crystallogr E Crystallogr Commun. 2015 May 13;71(Pt 6):o401–2. doi: 10.1107/S2056989015008658 (PMC4459327; doi:10.1107/S2056989015008658)

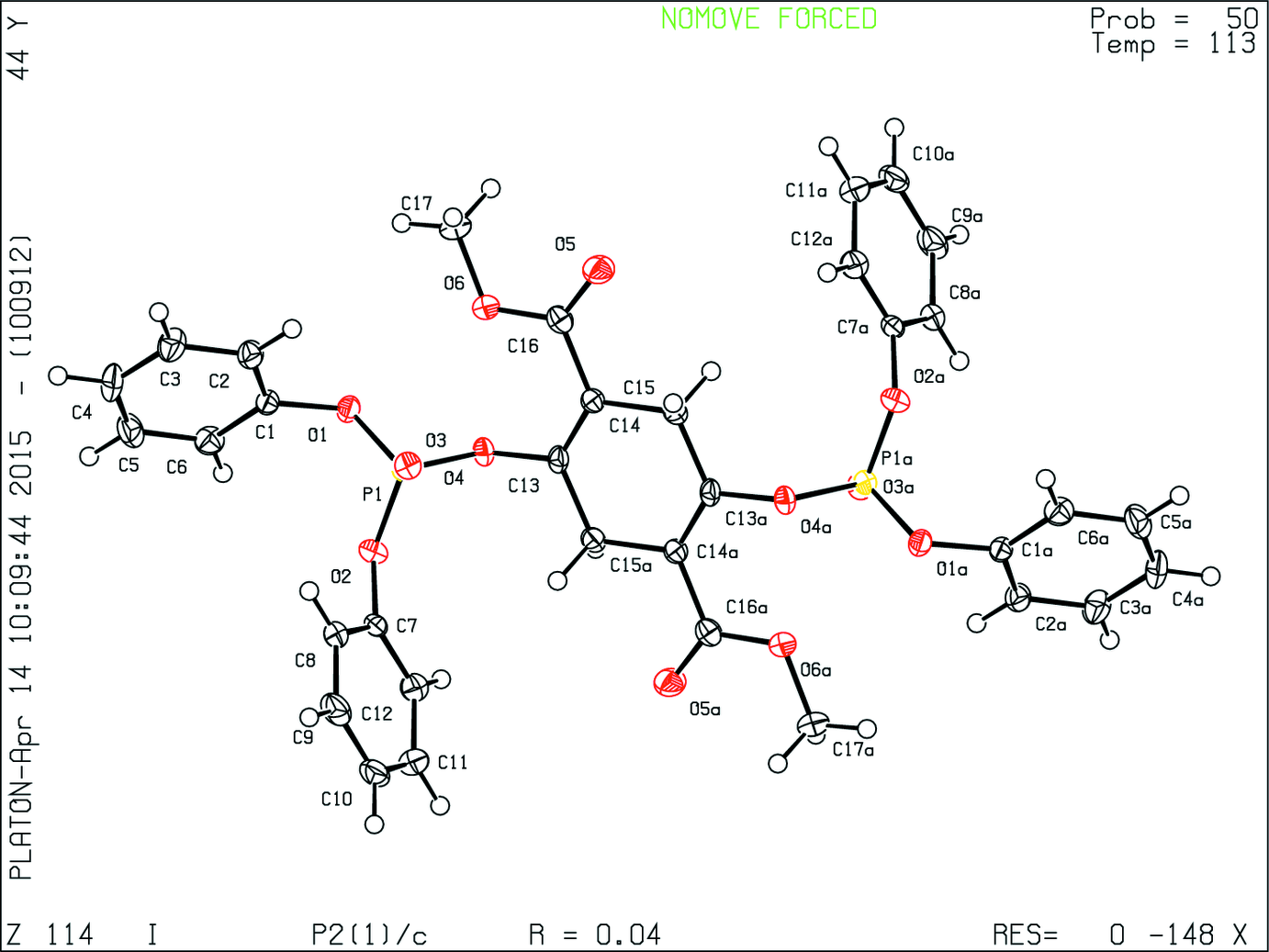

Supplement: Supplementary file 4 [file e-71-0o401-fig1.tif]

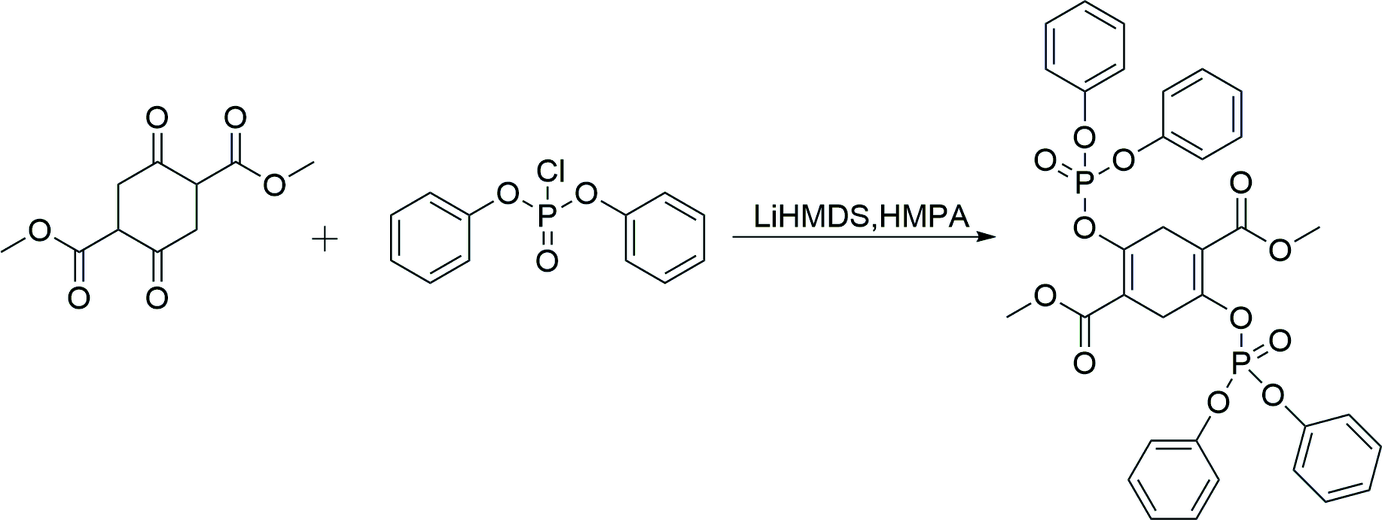

Supplement: Supplementary file 5 [file e-71-0o401-fig2.tif]
